# An unexpectedly large and loosely packed mitochondrial genome in the charophycean green alga *Chlorokybus atmophyticus*

**DOI:** 10.1186/1471-2164-8-137

**Published:** 2007-05-30

**Authors:** Monique Turmel, Christian Otis, Claude Lemieux

**Affiliations:** 1 Département de biochimie et de microbiologie, Université Laval, Québec, QC, G1K 7P4, Canada

## Abstract

**Background:**

The Streptophyta comprises all land plants and six groups of charophycean green algae. The scaly biflagellate *Mesostigma viride *(Mesostigmatales) and the sarcinoid *Chlorokybus atmophyticus *(Chlorokybales) represent the earliest diverging lineages of this phylum. In trees based on chloroplast genome data, these two charophycean green algae are nested in the same clade. To validate this relationship and gain insight into the ancestral state of the mitochondrial genome in the Charophyceae, we sequenced the mitochondrial DNA (mtDNA) of *Chlorokybus *and compared this genome sequence with those of three other charophycean green algae and the bryophytes *Marchantia polymorpha *and *Physcomitrella patens*.

**Results:**

The *Chlorokybus *genome differs radically from its 42,424-bp *Mesostigma *counterpart in size, gene order, intron content and density of repeated elements. At 201,763-bp, it is the largest mtDNA yet reported for a green alga. The 70 conserved genes represent 41.4% of the genome sequence and include *nad10 *and *trnL*(gag), two genes reported for the first time in a streptophyte mtDNA. At the gene order level, the *Chlorokybus *genome shares with its *Chara*, *Chaetosphaeridium *and bryophyte homologues eight to ten gene clusters including about 20 genes. Notably, some of these clusters exhibit gene linkages not previously found outside the Streptophyta, suggesting that they originated early during streptophyte evolution. In addition to six group I and 14 group II introns, short repeated sequences accounting for 7.5% of the genome were identified. Mitochondrial trees were unable to resolve the correct position of *Mesostigma*, due to analytical problems arising from accelerated sequence evolution in this lineage.

**Conclusion:**

The *Chlorokybus *and *Mesostigma *mtDNAs exemplify the marked fluidity of the mitochondrial genome in charophycean green algae. The notion that the mitochondrial genome was constrained to remain compact during charophycean evolution is no longer tenable. Our data raise the possibility that the emergence of land plants was not associated with a substantial gain of intergenic sequences by the mitochondrial genome.

## Background

Green algae belonging to the class Charophyceae gave rise to all land plant species [1,2]. In contrast to the large diversity of land plants, only a few thousand charophycean species are living today. Charophycean green algae and land plants form the lineage Streptophyta [3], whereas all of the other extant green algae belong to the sister lineage Chlorophyta [2]. Six monophyletic groups are currently recognized in the Charophyceae: the Mesostigmatales [4] represented by the scaly biflagellate *Mesostigma viride *that has long been regarded as a member of the Prasinophyceae (the earliest-diverging lineage of the Chlorophyta) [5], the Chlorokybales represented as well by a single species (*Chlorokybus atmophyticus*), the Klebsormidiales, the Zygnematales, the Coleochaetales and the Charales [6]. A phylogeny based on the nuclear 18S rRNA gene, the chloroplast *atpB *and *rbcL *and the mitochondrial *nad5 *from eight land plants, 26 charophycean taxa and five chlorophytes supports the notions that the Charales are sister to land plants and that charophycean green algae evolved progressively toward a more elaborated cellular complexity, occurring sequentially as biflagellated unicells, cubical packets of a few non-flagellated cells (sarcinoid morphology), unbranched/branched filaments and complex branched thalli with parenchymatous tissue [4,7]. In this four-gene tree, the deepest branch is occupied by the Mesostigmatales, the Chlorokybales emerge just after the Mesostigmatales, the Zygnematales are resolved as the next divergence and finally, the Coleochaetales are sister to the clade uniting the Charales and land plants. This branching order of charophycean lineages, however, is not entirely congruent with phylogenetic studies based exclusively on concatenated organelle genes [8-12]; in particular, the position of the Mesostigmatales and the identity of the charophycean lineage(s) that is sister to land plants have raised controversies.

The debate about the position of the Mesostigmatales has now been resolved [13,14]. The biflagellate *Mesostigma *represented the earliest divergence of the Streptophyta in the four-gene tree [4] and in single-gene trees based on 18S rDNA [15] and actin genes [16], whereas separate phylogenetic analyses of large data sets containing concatenated chloroplast genes/proteins [8,9,17,18] or mitochondrial proteins [10] placed the Mesostigmatales before the split of the Streptophyta and Chlorophyta. Because the latter phylogenies included members of only a few green plant lineages, sparse taxon sampling was suspected to be a possible cause of the conflicting results. We recently generated and annotated the *Chlorokybus *chloroplast genome sequence and in the course of comparing this sequence with all previously sequenced chloroplast genomes, gathered compelling evidence for the affiliation of *Mesostigma *with the Streptophyta [13]. This charophycean chloroplast genome remarkably resembles its *Mesostigma *homologue at various levels (size, gene content, gene order and intron content) and interestingly, the Mesostigmatales and Chlorokybales form a robust clade representing the deepest branch of the Streptophyta in phylogenies based on concatenated gene sequences. Strong support for *Mesostigma *being nested within the Streptophyta also comes from phylogenetic analyses of 125 nuclear-encoded proteins [14]; however, in this investigation, the relationship of the Mesostigmatales with the other charophycean groups could not be addressed because the *Closterium peracerosum*-*strigosum*-*littorale *complex (Zygnematales) was the only additional streptophyte alga examined.

In the present study, we undertook the sequencing of *Chlorokybus atmophyticus *mitochondrial DNA (mtDNA) with the goal of gaining better insight into the nature of the mitochondrial genome in the last common ancestor of all streptophytes. Given the abovementioned evidence for the close affiliation of *Chlorokybus *and *Mesostigma *in chloroplast phylogenomic trees, it was also of great interest to find out to what degree the *Chlorokybus *genome is similar to its *Mesostigma *counterpart and to mtDNAs of other charophycean green algae and land plants (in particular, bryophytes). Aside from *Mesostigma *[10], *Chaetosphaeridium globosum *(Coleochaetales) and *Chara vulgaris *(Charales) are the only charophyceans whose complete mtDNA sequences have been reported to date [19,20] and compared with their homologues in the liverwort *Marchantia polymorpha *[21] and the recently investigated moss *Physcomitrella patens *[22]. The three charophycean genomes are more densely packed with genes than their bryophyte homologues, although all five genomes have a similar coding potential. The higher degree of compaction observed for charophycean genomes largely explains their smaller sizes compared to land plant genomes. These observations are consistent with the notions that the last common ancestor of all streptophytes carried a relatively small and gene-rich mitochondrial genome and that substantial expansion of the intergenic spacers coincided with the emergence of land plants. Strikingly, at the gene organizational level, the *Mesostigma *genome has retained only traces of the numerous ancestral gene clusters preserved in other streptophyte mtDNAs. Gene order and intron content in the *Chara *genome most resemble those of land plant mtDNAs.

We report here that the *Chlorokybus *mitochondrial genome differs dramatically from *Mesostigma *mtDNA at the levels of size, gene order and intron content. Although the *Chlorokybus *genome is substantially larger than any other green algal mtDNA characterized yet, its gene organization features a pronounced degree of ancestral features. Our findings provide new light into mitochondrial genome evolution in the Streptophyta.

## Results

### General genomic features

**Figure 1:**
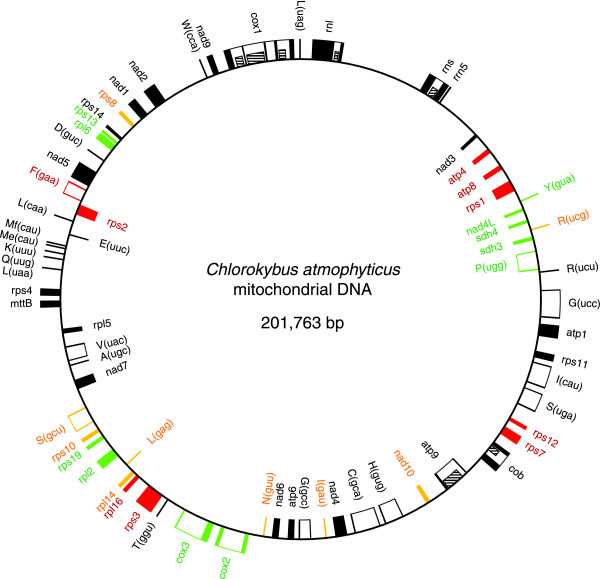
**Gene map of *Chlorokybus *mtDNA**. Genes (closed boxes) shown on the outside of the map are transcribed in a clockwise direction, whereas those on the inside of the map are transcribed counterclockwise. Introns are represented by open boxes and intron ORFs are denoted by narrow, hatched boxes. Blocks of gene sequences colinear with *Physcomitrella *mtDNA are shown as alternating series of green and red boxes. Genes absent from *Physcomitrella *mtDNA are represented in orange, whereas genes present in *Physcomitrella *mtDNA but located outside the conserved blocks are shown inblack. tRNA genes are indicated by the one-letter amino acid code (Me, elongator methionine; Mf, initiator methionine) followed by the anticodon in parentheses.

The *Chlorokybus *mtDNA sequence maps as a circular molecule of 201,763 bp (Figure 1), with an overall A+T content of 60.2%. This sequence is 4.8-fold larger than its *Mesostigma *counterpart [10] and is the largest among all green algal mitochondrial genomes sequenced to date. While the *Chlorokybus *mitochondrial genome is slightly smaller (20 kb) than the mtDNA of rapeseed [23] and is nearly two- to three-fold smaller than all six other angiosperm mtDNAs whose complete sequences have been reported thus far [24-29], its size exceeds those observed for the mtDNAs of the liverwort *Marchantia *[21] and the moss *Physcomitrella *[22] (Table 1). Considering that this charophycean green algal genome encodes 70 conserved genes, its coding capacity closely matches those of its homologues in the charophyceans *Chaetosphaeridium *(Coleochaetales) [19] and *Chara *(Charales) [20], the bryophytes *Marchantia *and *Physcomitrella *[21,22] (Table 1) and the chlorophyte *Nephroselmis olivacea *(Prasinophyceae) [30]. The gene repertoires of angiosperm mtDNAs (see Table 1 in [20]) and other completely sequenced green algal mtDNAs (see Table 1 in [31]) are more limited, featuring 12–61 conserved genes. The conserved genes in *Chlorokybus *mtDNA represent 41.4% of the total genome sequence, a coding density lower than those observed for *Marchantia *and *Physcomitrella *mtDNAs (Table 1). Ranging from 7 to 11,340 bp, the intergenic spacers in the *Chlorokybus *genome have an average size of about 1,690 bp. Repeated elements populate these regions, with the relative abundance of such elements in the genome (7.5%) being similar to that observed in *Marchantia *mtDNA (Table 1). With six group I introns and 14 group II introns, the *Chlorokybus *mitochondrial genome is richer in introns than its *Mesostigma *and *Chaetosphaeridium *counterparts (Table 1). All group I introns, with a single exception, contain an open reading frame (ORF), whereas only two group II introns feature an ORF (Figure 1).

### Gene content

The *Chlorokybus *mitochondrial genome encodes three rRNAs, 28 tRNAs, 17 ribosomal proteins, 21 ATP synthase and respiratory chain components and a protein involved in the Sec-independent translocation pathway (MttB). The 5S rRNA lacks the atypical insertion of 26–27 nucleotides observed in the variable loop B of its *Mesostigma *counterpart [10]. As reported for all streptophyte mtDNAs sequenced thus far, the set of encoded tRNA species is not sufficient to decode all of the 61 codons identified in the genome, owing to the absence of one or more species that can recognize the ACR codons specifying threonine.

The *nad10 *and *trnL*(gag) genes have not been previously identified in streptophyte mitochondria (Table 2); however, these genes have been reported in members of the Prasinophyceae, the earliest-diverging class of chlorophyte algae. Both genes are present in *Ostreococcus tauri *[32]; in addition, *nad10 *has been found in the prasinophyte *Nephroselmis *[30] and also in the cryptophyte alga *Rhodomonas salina *[33]. The gene repertoire of *Chlorokybus *is most similar to those of *Mesostigma *and *Chaetosphaeridium *(Table 2); in total, seven gene differences distinguish the latter two algae from *Chlorokybus*. The mitochondrial gene distribution among streptophytes highlights no specific alliance between *Mesostigma *and *Chlorokybus*, *i.e*. there are no genes that are uniquely missing or present in *Chlorokybus *and *Mesostigma *compared to their streptophyte counterparts (Table 2).

Potential functions could be assigned to the proteins specified by the seven intron ORFs in *Chlorokybus *mtDNA; however, BLASTP searches using as queries the proteins predicted from the ten free-standing ORFs larger than 100 codons [Genbank:EF463011] revealed no significant sequence similarity with any previously reported proteins. We found that the ORFs located within the *atp9 *and *cox1 *group II introns (*orf845 *and *orf755*, respectively) each encode a retron-type reverse transcriptase/type II intron maturase, whereas all five group I intron ORFs encode putative LAGLIDADG homing endonucleases. The protein specified by the *rnl *intron ORF (*orf170*) carries a single copy of the LAGLIDADG motif, whereas the products of the remaining group I intron ORFs (*orf260, orf274, orf296 *and *orf301*) feature two copies of this motif. Endonuclease activity specific for the cognate intron insertion site has been demonstrated for the homologues of three *Chlorokybus *intron ORFs located in positionally homologous introns in chlorophyte organelle genomes, *i.e*. for I-CsmI [34], I-CreI [35] and I-CpaII [36].

### Genome organization

At the level of gene organization, the *Chlorokybus *mitochondrial genome differs remarkably from its *Mesostigma *counterpart and unlike the latter displays significant similarity with *Chaetosphaeridium *and *Chara *mtDNAs as well as with the mitochondrial genomes of the bryophytes *Marchantia *and *Physcomitrella *(Table 3). Only four of the conserved genes in the *Mesostigma *genome are arranged in the same order in the *Chlorokybus *genome, forming two conserved gene clusters (*rpl6-rps13 *and *rps12-rps7*) that are vestiges of operons found in the bacterial ancestors of mitochondria. These two pairs of ribosomal protein genes also entirely account for the conservation of gene order observed between the *Mesostigma *genome and those of *Chaetosphaeridium*, *Marchantia *and *Physcomitrella*.

In contrast, a total of 20 to 22 genes forming eight to ten conserved gene clusters account for the conservation of gene order observed between the *Chlorokybus *genome and its homologues in the Coleochaetales, Charales and bryophytes (Table 3). In Figure 1 are highlighted the eight clusters that the *Chlorokybus *genome shares with its homologue in the moss *Physcomitrella*. These conserved clusters, which encode about one third of the genes common to these genomes (20/61), comprise six gene pairs, one triplet and one quintuplet. All gene pairs, except *rps2-trnF*(gaa), have been identified in the mtDNAs of chlorophytes [30,37] or other eukaryotes, including the mtDNA of the heterotrophic jakobid flagellate *Reclinomonas americana *[38]. The two other clusters have been detected only as fragments (*sdh3-sdh4-nad4L, rps1-atp8 *and *atp8-atp4*) in these genomes.

The *Chara *genome is the charophycean green algal mtDNA that most closely resembles its land plant counterparts, with more than 86% of the genes common to *Chara *and the two investigated bryophytes being part of conserved clusters. The smaller number of clusters identified in the *Chara*/*Physcomitrella *comparison (10 clusters) versus the *Chara*/*Marchantia *comparison (16 clusters) indicates that more rearrangements occurred in the lineage leading to the liverwort *Marchantia *than in that leading to *Physcomitrella*. Using GRIMM, we estimated that only 12 inversions would be required to interconvert the mitochondrial gene orders of *Chara *and *Physcomitrella *(Table 3).

### Introns

**Figure 2:**
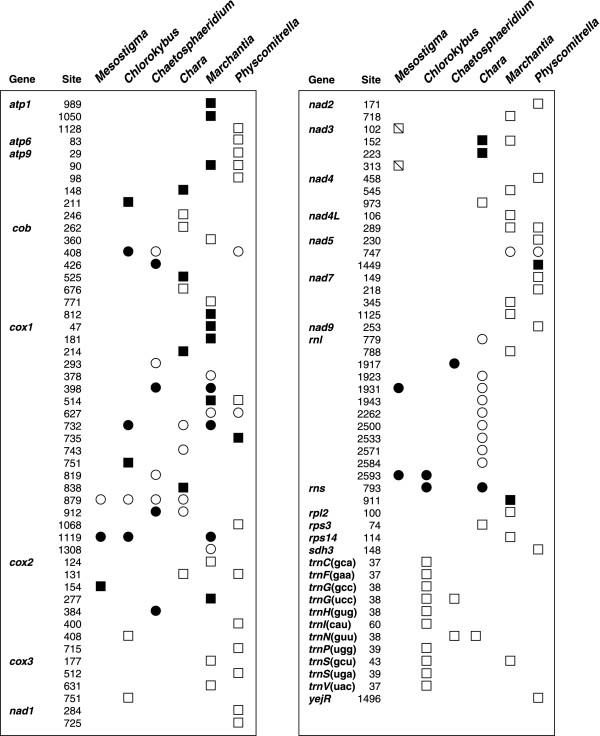
**Distribution of introns in *Chlorokybus *and other streptophyte mtDNAs**. Circles denote the presence of group I introns and squares denote the presence of group II introns. Divided squares represent *trans*-spliced group II introns. Open symbols denote the absence of intron ORFs, whereas filled symbols denote their presence. Intron insertion sites in genes coding for tRNAs and proteins are given relative to the corresponding genes in *Reclinomonas americana *mtDNA; insertion sites in *rns *and *rnl *are given relative to *Escherichia coli *16S and 23S rRNAs, respectively. For each insertion site, the position corresponding to the nucleotide immediately preceding the intron is reported.

The six group I introns in *Chlorokybus *mtDNA reside in *cob*, *cox1 *(sites 732, 879 and 1119), *rns *and *rnl *at the same gene locations as structurally similar mitochondrial introns previously reported in charophycean green algae and bryophytes (Figure 2). Most of these introns have also homologues in chlorophyte mtDNAs [30,31,37,39,40]. The *Chlorokybus cox1 *intron at site 732 has homologues not only in *Chara*, *Marchantia*, chlorophytes and land plants but also in fungi [20]. This is the insertion site of the mobile intron that angiosperms acquired on multiple occasions [41]. Like its land plant, fungal and chlorophyte counterparts, the site-732 *Chlorokybus cox1 *intron encodes a double LAGLIDADG homing endonuclease; however, it shares no specific relationship with fungal introns. The maximum likelihood (ML) and maximum parsimony (MP) trees inferred from the site-732 *cox1 *intron sequences of 15 green algal/land plant taxa and four fungi (169 sites corresponding to the intron core) were congruent in showing that the *Chlorokybus *intron affiliates with its *Chara *and *Marchantia *counterparts and that the resulting clade occupies a sister position relative to that containing the introns from the chlorophytes *Prototheca wickerhamii *and *Chlorella vulgaris *(data not shown).

Four of the group II introns in *Chlorokybus *mtDNA lie in protein-coding genes (*atp9*, *cox1*, *cox2 *and *cox3*), whereas the remaining ten introns reside in tRNA genes (Figure 2). Only two of these introns, those in *trnG*(ucc) and *trnS*(gcu), are known to have homologues at identical positions in the corresponding mitochondrial genes of other charophycean green algae and/or land plants (Figure 2). In the case of the ORF-containing *cox1 *intron at site 879, positional and structural homologues carrying a similar ORF have been identified exclusively outside the Streptophyta, *i.e*. in the chlorophyte *Oltmannsiellopsis viridis *[31], the brown alga *Pylaiella littoralis *[42] and the cryptophyte alga *Rhodomonas salina *[33]. Unlike the *orf606 *of its *Oltmannsiellopis *counterpart [31], the *orf755 *of the *Chlorokybus cox1 *intron can be aligned with the entire *Rhodomonas cox1 *intron ORF (*orf762*). The majority of introns in *Chlorokybus *tRNA genes are inserted two nucleotides upstream of the anticodon; only the *trnS*(gcu) and *trnI*(cau) introns represent notable exceptions. Instead of being located in the anticodon loop, the insertion site of the *trnS*(gcu) intron lies within the anticodon arm (six nucleotides upstream of the anticodon), whereas that of the *trnI*(cau) intron occurs in the TψC arm. In this context, it is interesting to mention that, in the chloroplast genomes of land plants and charophycean green algae belonging to the Charales, Coleochaetales and Zygnematales, a number of group II introns in tRNA genes are also inserted two nucleotides upstream of the anticodon [11]. Only the streptophyte chloroplast introns found in *trnV*(uac) occur at the same gene location as one of the *Chlorokybus *mitochondrial group II introns. Despite this positional homology, our sequence comparisons uncovered no specific relationship between the *Chlorokybus *mitochondrial *trnV*(uac) intron and its chloroplast counterparts.

### Repeated elements

As estimated with RepeatMasker, repeats represent 7.5% of the *Chlorokybus *mitochondrial genome sequence (Table 1). Although there is a similar proportion of repeats in *Marchantia *mtDNA, such sequences are less abundant in the mitochondria of the moss *Physcomitrella *and the three other charophycean green algal mitochondrial genomes investigated to date (Table 1). The *Chlorokybus *repeats are generally located in intergenic regions and comprise both tandem and dispersed sequence elements.

In the case of the tandem repeats, 11 different repeat units ranging from 4 to 28 bp in size and present in 2 to 39 copies per locus were identified (see Additional file 1: Supplementary Table 1 for a list of the tandem repeat units and a description of their features). The tandem repeats made up of units smaller than 11 bp are dispersed in the *Chlorokybus *genome, occurring at up to 249 distinct loci. With regard to the dispersed repeats, 11 different units were identified (see Additional file 1: Supplementary Table 2 for the sequences of the dispersed repeat units), with at least two (AATGCA and GGGCTGC) being clearly related to some of the repeat units constituting the tandem repeats (ATGCA, TGCA, GGGCTGCACT and GGGCT). The sizes of the units featured by the dispersed repeats range from 6 to 8 bp and the numbers of perfectly identical copies vary from 29 to 520 (see Additional file 1: Supplementary Table 2 for the copy numbers of the dispersed repeat units). In addition to the latter copies of dispersed repeat units, we find a large number of copies carrying a single mismatch in their sequences. Similar or distinct repeat units often associate to form longer repeats, including stem-loop structures. None of the repeated units reported here were found to be identical to those present in the *Chlorokybus *chloroplast genome [13].

### Phylogenetic analyses

**Figure 3:**
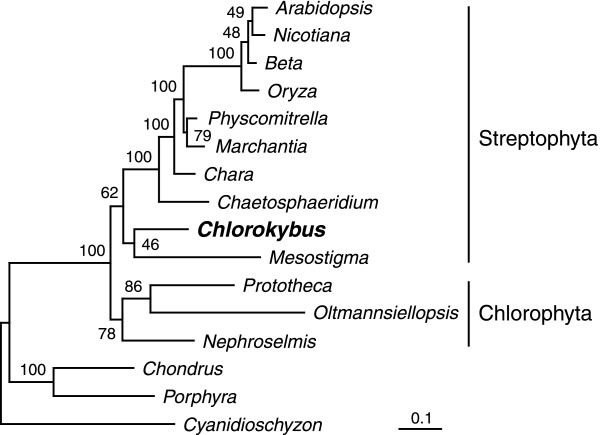
**Phylogenetic position of *Chlorokybus *within the Viridiplantae**. Best ML tree inferred from 18 mitochondrial-encoded proteins shared by 16 algal/plant taxa. The three non-green algae (bottom of the figure) were used as outgroup. Bootstrap values obtained in PHYML analyses are indicated on the corresponding nodes. The detailed names of taxa are reported in the Methods. The 18 genes analyzed are as follows: *atp4,6,8,9, cob, cox1,2,3, mttB, nad1,2,3,4,4L,5,6, rps3,12*.

To determine the phylogenetic position of *Chlorokybus *as inferred from mitochondrial genomic data, we analyzed an amino acid data set containing a total of 4,024 sites using the ML method (Figure 3). This data set was derived from 18 protein-coding genes common to the mtDNAs of 13 green algal/land plant taxa and three non-green algae. As expected, ML analysis strongly supported the placement of *Chlorokybus *within the Streptophyta; however, the precise relationship of this charophycean green alga with *Mesostigma *could not be determined with confidence. The best ML tree identified a weakly supported clade uniting *Chlorokybus *and *Mesostigma *at the base of the Streptophyta. In alternative tree topologies, *Mesostigma *was found either as sister to all streptophytes or before the divergence of the Streptophyta and Chlorophyta. The branch leading to *Mesostigma *is markedly longer than that leading to *Chlorokybus*, suggesting that long-branch attraction artefacts are responsible for the positioning of the former alga outside the Streptophyta. Consistent with this notion, MP analysis, which is known to be more sensitive to long-branch attraction [43], provided very strong support (93% bootstrap support) for *Mesostigma *representing the earliest-diverging lineage of the Viridiplantae (data not shown).

## Discussion

### Highly variable gene density in charophycean mitochondrial genomes

The large size and spacious intergenic spacers of the *Chlorokybus *mitochondrial genome represent unusual traits. At 201,763 bp, this 70-gene encoding mtDNA is three- to five-fold larger than the three other previously characterized charophycean genomes, all of which have approximately the same number of conserved genes (Table 1). Moreover, it is about twice as large as its homologue carrying 57 conserved genes in the chlorophyte *Pseudendoclonium akinetum *(Ulvophyceae), the largest green algal mitochondrial genome sequence reported so far [40]. With intergenic spacers accounting for 41% of its sequence, *Chlorokybus *mtDNA is now also recognized as the most loosely packed green algal mitochondrial genome (Table 1). Of particular interest is the close resemblance of this charophycean genome with the mtDNA of the bryophyte *Marchantia *with regards to size, gene content, gene density and abundance of repeats [21]. As is the case for bryophytes, the intergenic sequences in *Chlorokybus *mtDNA have no recognizable homology to any known sequences in public databases, suggesting that the increased size of these regions is largely accounted for by expansion of endogenous sequences. In angiosperms, a significant fraction of the mtDNA size (*Arabidopsis*, 1.1% [27]; sugar beet, 2.1% [24]; tobacco, 2.5% [26]; wheat, 3.0% [29]; rapeseed, 3.6% [23]; maize, 4.4% [28]; rice, 6.3% [25]) is accounted for by sequences derived from the chloroplast genome. The important variation in gene density reported here thus indicates that mitochondrial genome evolution in charophycean green algae is less uniform than previously thought. This unexpected finding challenges current concepts that the mitochondrial genome was constrained to remain compact during the evolution of charophycean green algae and that this evolutionary pressure became relaxed when land plants emerged [20]. Two waves of mitochondrial genome expansion have been documented during land plant evolution: one coinciding with the transition from charophycean green algae to land plants and the other with the emergence of angiosperms [20,44].

The prominent size and low gene density of the *Chlorokybus *genome sequence are compatible with two evolutionary scenarios. First, as suggested earlier [20], it is possible that the mitochondrial genome of the last common ancestor of all streptophytes featured a very compact gene organization such as those observed in the *Mesostigma *and *Chara *genomes and that intergenic regions enlarged independently and convergently in the basal charophycean lineage leading to *Chlorokybus *and the late-diverging streptophyte lineage leading to bryophytes. Genome expansion in specific lineages has also been proposed to explain the atypical mtDNA size and relatively low gene density observed for the ulvophyte *Pseudendoclonium *[40]. In agreement with this notion, the more compact 56,761-bp mtDNA of *Oltmannsiellopsis viridis *[31], a representative of a separate early-diverging lineage of the Ulvophyceae, falls within the size range observed for members of the three other chlorophyte classes [30,32,37,45]. Although the abovementioned scenario for streptophyte mtDNA evolution is supported by the finding of densely packed mtDNAs in most investigated chlorophytes (see Table 1 in reference [31]), other algae [33,42,46-49] and unicellular eukaryotes from other lineages [50], the currently available distribution of gene density for charophycean mitochondrial genomes does not rule out the possibility that the ancestral streptophyte mitochondrial genome was less compact than previously assumed and that intergenic spacers contracted independently in the lineages leading to *Mesostigma *and *Chara*. If correct, this second evolutionary scenario predicts that the emergence of the earliest diverging land plants was not necessarily accompanied with a substantial gain of sequences. Mitochondrial genome sequences from more charophycean green algae, in particular from members of the Klebsormidiales, Zygnematales and additional lineages from the Coleochaetales will be required to fully understand the dynamics of mitochondrial genome evolution in this algal group.

### Contrasting evolutionary trends of the mitochondrial genome in the *Chlorokybus *and *Mesostigma *lineages

Our recent analysis of three distinct sets of chloroplast genome data (gene order, gene content and sequences of concatenated genes) revealed that *Chlorokybus *is closely related to *Mesostigma *[13]. In light of this close relationship, our finding that the *Chlorokybus *mitochondrial genome differs considerably from its *Mesostigma *counterpart not only in gene density but also in gene order came as a surprise. No specifically shared genomic features could be identified in these charophycean mitochondrial genomes. On the other hand, the substantial differences in intron content displayed by *Chlorokybus *and *Mesostigma *mtDNAs were anticipated considering that the variable intron distributions previously reported for charophycean algae and bryophytes are consistent with numerous gains of lineage-specific introns by each genome (see Figure 2 and [20,22]). In this context, it should be mentioned that the mobile group I introns found at site 2593 within the *Chlorokybus *and *Mesostigma rnl *genes (Figure 2) cannot be necessarily regarded as a shared character, as an homologous intron is present at the same location in *Nephroselmis *mtDNA [30]. The presence of a large number of group II introns in tRNA genes is unique to the *Chlorokybus *mitochondrial genome. Because eight of these ten tRNA introns lie at the same site within the anticodon loop, it is possible that they arose through proliferation of a founding intron in the *Chlorokybus *lineage (perhaps the *trnG*(ucc) intron that is also found in *Chaetosphaeridium *mtDNA) and that they diverged substantially in sequence following their insertion in different tRNA genes.

The *Chlorokybus *mitochondrial genome has retained numerous ancestral characters at the gene content and gene organizational levels. This genome is the most gene-rich among the green algal mtDNAs sequenced thus far and features two genes that have not been reported to be mtDNA-encoded in streptophytes prior to our study [*nad10 *and *trnL(gag)*], thus bringing to 75 the number of genes making up the gene repertoire of the common ancestor of all streptophytes (Table 2). Of these genes, seven (*rpl2, rps1, sdh3, sdh4, yejR, yejU *and *yejV*) are not found in the Chlorophyta. The mitochondrial genome sequences currently available for chlorophytes suggest that the gene repertoire of the common ancestor of these algae was more limited and included 70 genes, only two of which are not present in the Streptophyta [*rnpB *and *trnT*(ugu)].

Like its bryophyte counterparts, the *Chlorokybus *mitochondrial genome has maintained several ancestral gene clusters even though short repeated sequences and spacious intergenic regions increase opportunities for gene rearrangements [44,51]. On the other hand, clear evidence that rearrangements affected a few ancestral clusters comes from the observation that the *Chlorokybus *genome has not preserved the eubacteria-like arrangement of the ribosomal protein genes corresponding to the contiguous *S10*, *spc *and α operons of *Escherichia coli *(the cluster corresponding to the 11-gene segment delimited by *rps10 *and *rps11 *in *Marchantia*) as well as the *nad5*-*nad4*-*nad2 *cluster, both of which are found in the *Chaetosphaeridium*, *Chara *and the two bryophyte genomes. Interestingly, some of the eight to ten clusters shared by *Chlorokybus *and other streptophyte mtDNAs (Table 3) exhibit gene linkages [*rps2*-*trnF*(gga), *trnP*(ugg)-*sdh3 *and *nd4L*-*trnY*(gua)] that have not been documented outside the Streptophyta, suggesting that they arose very early during streptophyte evolution.

Evidently, the *Chlorokybus *and *Mesostigma *mitochondrial genomes were shaped by divergent forces acting at multiple levels. First, let us consider the gene content and more particularly, the genes lost specifically in each of these charophycean lineages. The *Chlorokybus *gene repertoire lacks only one of the 65 genes present in *Mesostigma *mitochondria [*trnR*(acg)] and according to the gene distribution currently available for charophycean green algae and bryophytes (Table 2), this gene was lost uniquely in the *Chlorokybus *lineage. Aside from *nad10 *and *trnL*(gag), all but one (*rps8*) of the four mitochondrial genes that are present in *Chlorokybus *but missing from *Mesostigma *represent gene losses unique to the *Mesostigma *lineage (Table 2). With respect to gene organization, the *Mesostigma *mitochondrial genome shows even more differences with its *Chlorokybus *homologue. In sharp contrast to all other completely sequenced streptophyte mtDNAs, ancestral gene clusters are virtually absent from *Mesostigma *mtDNA (Table 3), implying that extensive gene rearrangements took place in this charophycean lineage. In fact, gene shuffling events were so extensive that they disrupted the continuity of the *nad3 *gene within the two group II introns, giving rise ultimately to the scattering of the three exons and *trans *splicing of the introns at the RNA level [10]. Considering that gene rearrangements in organelle genomes are often associated with repeated sequences [44,51], the absence of such elements from *Mesostigma *mtDNA might suggest that short repeats once existed as integral components of the mitochondrial genome but disappeared almost completely during streamlining of the intergenic regions. Gene rearrangements are not the only major, lineage-specific evolutionary events that marked the *Mesostigma *genome. In parallel, the rate of mtDNA sequence evolution accelerated in the *Mesostigma *lineage, leading to difficulties in correctly positioning this alga in mitochondrial phylogenomic trees (Figure 3).

The evolutionary pattern displayed by the *Mesostigma *mitochondrial genome is in some ways reminiscent of the 'reduced derived' pattern described for the mtDNAs of the chlorophyte green algae belonging to the Chlamydomonadales (Chlorophyceae) [39,52-54] as well as for the mtDNA of the enigmatic chlorophyte *Pedinomonas minor *[30]. The latter pattern is characterized by reduction of both genome size and gene content and by acceleration of primary sequence evolution [30,50]. The reduced derived mtDNAs completely sequenced thus far vary from 15 to 25 kb in size (see Table 1 in reference [31]), encode 12 to 22 genes and display a number of derived characters at the levels of their gene organization and structure. Clearly, the sequence acceleration reported here for the tightly packed *Mesostigma *mitochondrial genome is a genomic trait shared by all reduced derived mtDNAs. But whether the evolution of this trait was connected with events of genome reduction and limited gene losses in the *Mesostigma *lineage remains unknown.

## Conclusion

In uncovering remarkable differences in size and gene organization between the 201,763-bp *Chlorokybus *mitochondrial genome and its 42,424-bp *Mesostigma *counterpart, the study reported here provides new insights into mitochondrial genome evolution in the Streptophyta. Before this study, the prevailing view was that the ancestral streptophyte mitochondrial genome resembled the *Mesostigma *genome in being tightly packed with genes and that this trait was shared with other charophycean green algae. Our findings raise the possibility that the mitochondrial genome of the last common ancestor of all streptophytes bore resemblance to *Chlorokybus *and bryophyte mtDNAs with regard to size and gene density, implying that the emergence of land plants was not necessarily associated with a substantial gain of intergenic sequences. Mitochondrial genome sequences from a broader range of charophycean green algae will be required to determine whether the unusually large size of the *Chlorokybus *genome reflects an ancestral or a derived trait.

## Methods

### DNA cloning, sequencing and sequence analysis

*Chlorokybus atmophyticus *was obtained from the Sammlung von Algenkulturen Göttingen (SAG 48.80) and grown in medium C [55] under 12 h light/dark cycles. A random clone library was prepared from 1500- to 2000-bp fragments derived from a fraction containing both chloroplast DNA and mtDNA using the pSMART-HCKan (Lucigen Corporation, Middleton, WI) plasmid [20]. DNA templates were generated with the QIAprep 96 Miniprep kit (Qiagen Inc., Mississauga, Canada) and sequenced as described previously [56]. Sequences were edited and assembled using SEQUENCHER 4.2 (Gene Codes Corporation, Ann Arbor, MI). Genomic regions not represented in the clones analyzed were sequenced from PCR-amplified fragments. The fully annotated mitochondrial genome sequence has been deposited in [Genbank:EF463011].

Genes and ORFs were identified as described previously [57]. Introns were modelled according to the nomenclatures proposed for group I [58] and group II [59] introns. Homologous introns were identified by BLASTN searches [60] against the non-redundant database of National Center for Biotechnology Information. Repeated sequences were identified with REPuter 2.74 [61] using the -f (forward) and -p (palindromic) options at minimum lengths of 30 bp and were classified with the Comparative Repeat Analysis program [62]. Number of copies of each repeat unit was determined with FINDPATTERNS of the Wisconsin package version 10.3 (Accelrys, San Diego, CA, USA) or FUZZNUC in EMBOSS 2.9.0 [63]. Stem-loop structures and tandem repeats were identified using PALINDROME and ETANDEM in EMBOSS 2.9.0, respectively. Genomic regions containing non-overlapping repeated elements were identified with RepeatMasker [64] running under the WU-BLAST 2.0 [65] search engine.

### Analysis of genome rearrangements

A custom-built program (Patrick Charlebois, Claude Lemieux and Monique Turmel, unpublished data) was used to identify the gene clusters that are conserved in selected pairs of streptophyte mtDNAs. The numbers of gene permutations by inversions in pairwise mtDNA comparisons were inferred using the GRIMM web server [66]. In these analyses, the order of all genes/pseudogenes shared by each mtDNA pair was investigated. In the pairwise comparisons involving *Marchantia *mtDNA, one copy of the duplicated *trnMf*(cau) gene (the copy between *atp4 *and *rnl*) as well as one copy of the duplicated *trnY*(gua) [the copy between *trnR*(ucu) and *trnR*(acg)] were excluded from the data set.

### Phylogenetic analyses

GenBank files for the following mitochondrial genomes were retrieved: *Arabidopsis thaliana *[Genbank:NC_001284], *Beta vulgaris *[Genbank:NC_002511], *Chaetosphaeridium globosum *[Genbank:NC_004118], *Chara vulgaris *[Genbank:NC_005255], *Chondrus crispus *[Genbank:NC_001677], *Chlorokybus atmophyticus *[Genbank:EF463011], *Cyanidioschyzon merolae *[Genbank:NC_000887], *Marchantia polymorpha *[Genbank:NC_001660], *Mesostigma viride *[Genbank:NC_008240], *Nephroselmis olivacea *[Genbank:NC_008239], *Nicotiana tabacum *[Genbank:NC_006581], *Oltmannsiellopsis viridis *[Genbank:NC_008256], *Oryza sativa *[Genbank:NC_007886], *Physcomitrella patens *[Genbank:NC_007945], *Prototheca wickerhamii *[Genbank:NC_001613] and *Porphyra purpurea *[Genbank:NC_002007]. A data set of 18 concatenated protein sequences was derived as described previously [20] from all protein-coding genes shared by these genomes. Phylogenetic analyses of the data set were carried out using ML and MP methods. ML trees were computed with PHYML 2.4.5 [67] under the WAG+Γ+I model of amino acid substitutions and bootstrap support for each node was calculated using 100 replicates. MP trees were inferred using PROTPARS in PHYLIP 3.65 [68] and confidence of branch points was assessed by bootstrap percentages after 100 replications.

A data set of intron sequences inserted at site 732 within the *cox1 *gene (169 sites corresponding to unambiguously aligned regions of the intron core) was analyzed using ML and MP methods. These analyses were performed with PAUP*4.0b10 [69] using a uniform rate of substitutions across sites. ML trees were inferred under the HKY model. Bootstrap support was assessed after 100 replications.

## Authors' contributions

CL and MT conceived and designed the study, and wrote the manuscript. CL performed most of the sequence analyses and generated the figures. MT also contributed to the analysis and interpretation of the data. CO carried out the sequencing of the Chlorokybus mitochondrial genome, identified the repeated sequence elements and analyzed the introns in this genome. All authors read and approved the final manuscript.

## Supplementary Material

Additional file 1 **Supplementary tables**. Supplementary tables S1 and S2 report the features of the tandem and dispersed repeats in the *Chlorokybus *mitochondrial genome. Click here for file

## Figures and Tables

**Table 1:** General features of mtDNAs from *Chlorokybus*, other charophycean green algae and bryophytes

**Genome feature**	***Mesostigma***	***Chlorokybus***	***Chaetosphaeridium***	***Chara***	***Marchantia***	***Physcomitrella***
Size (bp)	42,424	201,763	56,574	67,737	186,609	105,340
A+T content (%)	67.8	60.2	65.6	59.1	57.6	59.4
Coding sequences (%) ^a^	85.0	41.4	71.4	90.8	50.2	65.9
Repeated sequences (%) ^b^	0.1	7.5	0.1	2.6	6.9	1.1
Gene content ^c^	65	70	67	68	69	65
Introns						
Group I	4	6	9	14	7	3
Group II						
*Cis*-spliced	1	14	2	13	25	24
*Trans*-spliced	2	0	0	0	0	0

^a ^Conserved genes (including pseudogenes), introns and intron ORFs were considered coding sequences.

^b ^Non-overlapping repeat elements were mapped on each genome with RepeatMasker using the repeats ≥ 30 bp identified with REPuter as input sequences.

^c ^Pseudogenes, unique ORFs and intron ORFs were not considered.

**Table 2:** Differences between the mitochondrial gene repertoires of *Chlorokybus*, other charophycean green algae and bryophytes

**Gene **^a^	***Mesostigma***	***Chlorokybus***	***Chaetosphaeridium***	***Chara***	***Marchantia***	***Physcomitrella***
*nad7*	+	+	+	+	- ^b^	+
*nad10*	-	+	-	-	-	-
*rpl2*	-	+	+	+	+	+
*rpl14*	+	+	-	+	-	-
*rps8*	-	+	-	-	+	-
*rps10*	+	+	+	+	+	-
*rps13*	+	+	+	-	+	+
*yejR*	-	-	-	+	+	+
*yejU*	-	-	-	+	+	+
*yejV*	-	-	-	+	+	+
*trnI*(cau)	-	+	+	+	+	+
*trnI*(gau)	+	+	+	+	-	-
*trnL*(caa)	+	+	+	- ^b^	+	+
*trnL*(gag)	-	+	-	-	-	-
*trnN*(guu)	+	+	+	+	+	-
*trnR*(acg)	+	-	+	+	+	+
*trnR*(ucg)	+	+	-	-	+	-
*trnS*(acu)	-	-	+	-	-	-
*trnS*(gcu)	+	+	+	+	+	-
*trnT*(ggu)	-	+	+	+	+	+

^a ^Only the conserved genes that are missing in one or more genomes are indicated. Plus and minus signs denote the presence and absence of genes, respectively. A total of 55 genes are shared by all compared mtDNAs: *atp1,4,6,8,9, cob, cox1,2 3, mttB, nad1,2 3, 4,4L,5,6,9, rnl, rns, rpl5,6,16, rps1,2,3,4,7,11,12,14,19, rrn5, sdh3,4, trnA*(ugc),*C*(gca),*D*(guc),*E*(uuc),*F*(gaa),*G*(gcc),*G*(ucc),*H*(gug), *K*(uuu),*L*(uaa),*L*(uag),*Me*(cau),*Mf*(cau),*P*(ugg),*Q*(uug),*R*(ucu),*S*(uga),*V*(uac),*W*(cca),*Y*(gua).

^b ^*trnL*(caa) and *nad7 *are pseudogenes in *Chara *and *Marchantia *mtDNAs, respectively.

**Table 3:** Conservation of gene order in streptophyte mitochondrial genomes

**Compared genomes**	**No. of conserved gene clusters **^a^	**No. of genes in clusters/no. of shared genes **^b^	**Similarity index **^c^	**No. of reversals **^d^
*Chara/Physcomitrella*	10	59/64	9.2	12
*Chaetosphaeridium/Physcomitrella*	10	40/62	6.5	26
*Marchantia/Physcomitrella*	14	59/65	6.5	14
*Chaetosphaeridium/Chara*	12	43/65	5.5	28
*Chara/Marchantia*	16	58/67	5.4	20
*Chaetosphaeridium/Marchantia*	15	46/65	4.7	27
*Chlorokybus/Physcomitrella*	8	20/61	4.1	47
*Chlorokybus/Chaetosphaeridium*	9	22/65	3.8	49
*Chlorokybus/Chara*	9	21/65	3.6	51
*Mesostigma/Physcomitrella*	2	4/61	3.3	57
*Chlorokybus/Marchantia*	10	21/66	3.2	51
*Mesostigma/Chaetosphaeridium*	2	4/65	3.1	60
*Mesostigma/Chara*	2	4/65	3.1	61
*Mesostigma/Marchantia*	2	4/65	3.1	60
*Chlorokybus/Mesostigma*	2	4/66	3.0	63

^a ^A conserved gene cluster is defined here as a suite of genes/gene pieces with the same order and polarities in the pair of compared genomes.

^b ^The ratio of these two values represents the fraction of shared genes/gene pieces found in conserved clusters.

^c ^This value was obtained by dividing the percentage of shared genes/gene pieces found in conserved clusters by the number of conserved clusters.

^d ^GRIMM was used to infer the numbers of reversals required to transform gene order in the compared genomes.
