# Supplementary material for: An unexpectedly large and loosely packed mitochondrial genome in the charophycean green alga Chlorokybus atmophyticus
Source: BMC Genomics. 2007 May 30;8:137. doi: 10.1186/1471-2164-8-137 (PMC1894977; doi:10.1186/1471-2164-8-137)
Supplement: Additional file 1 — Supplementary tables. Supplementary tables S1 and S2 report the features of the tandem and dispersed repeats in the Chlorokybus mitochondrial genome. [file 1471-2164-8-137-S1.pdf]

Table S1: Tandem repeats in the *Chlorokybus* mitochondrial genome

Repeat unit	Size (bp)	No. of units/site [a]	No. of sites	Total no. of units
TGCA	4	2–15	249	612
ATGCA	5	2–39	105	383
ATTCC	5	2–11	27	66
GGGCT	5	2–12	81	204
GCACT	5	2–34	111	356
CAAAG	5	2–16	35	86
CCCAA	5	2–10	26	61
CCCAAA	6	2–13	2	15
GGGCTGCACT	10	2–4	11	28
TTCAAATTCAAGAA	14	6	1	6
TACCACGAAGTGGTAGGATTCGAAGAAT	28	11	1	11

[a] The number of units was estimated by allowing one substitution per repeat unit.

Table S2: Dispersed repeats in the *Chlorokybus* mitochondrial genome

Repeat unit	Size (bp)	Copy number	
		Perfect match	One mismatch
AATGCA	6	520	4845
CGGAGA	6	64	1020
TGGCTCT	7	26	410
TGCGCCT	7	91	439
GACCAAG	7	30	338
GTAAGAG	7	43	578
GTGGTAG	7	216	729
GGGCTGC	7	214	1051
TAATCGA	7	292	1851
GCTGCAGC	8	140	1018
GGCTTCCA	8	29	113
